# Spatially Resolved Temperature Distribution in a Rare-Earth-Doped Transparent Glass-Ceramic

**DOI:** 10.3390/s22051970

**Published:** 2022-03-02

**Authors:** Ivan Sedmak, Rok Podlipec, Iztok Urbančič, Janez Štrancar, Michel Mortier, Iztok Golobič

**Affiliations:** 1 Laboratory for Thermal Technology (LTT), Faculty of Mechanical Engineering, University of Ljubljana, 1000 Ljubljana, Slovenia; iztok.golobic@fs.uni-lj.si; 2 Ion Beam Center, Helmholtz-Zentrum Dresden-Rossendorf e.V., Bautzner Landstrasse 400, 01328 Dresden, Germany; rok.podlipec@ijs.si; 3 Laboratory of Biophysics, Department of Solid State Physics, Jožef Stefan Institute, 1000 Ljubljana, Slovenia; iztok.urbancic@ijs.si (I.U.); janez.strancar@ijs.si (J.Š.); 4 Chimie ParisTech, Institute de Recherche de Chimie Paris, PSL Research University, 75005 Paris, France; michel.mortier@chimieparistech.psl.eu

**Keywords:** temperature-dependent fluorescence, co-doped glass-ceramic, axial temperature distribution, micro-scale temperature measurements

## Abstract

Knowing the temperature distribution within the conducting walls of various multilayer-type materials is crucial for a better understanding of heat-transfer processes. This applies to many engineering fields, good examples being photovoltaics and microelectronics. In this work we present a novel fluorescence technique that makes possible the non-invasive imaging of local temperature distributions within a transparent, temperature-sensitive, co-doped Er:GPF1Yb0.5Er glass-ceramic with micrometer spatial resolution. The thermal imaging was performed with a high-resolution fluorescence microscopy system, measuring different focal planes along the *z*-axis. This ultimately enabled a precise axial reconstruction of the temperature distribution across a 500-µm-thick glass-ceramic sample. The experimental measurements showed good agreement with computer-modeled heat simulations and suggest that the technique could be adopted for the spatial analyses of local thermal processes within optically transparent materials. For instance, the technique could be used to measure the temperature distribution of intermediate, transparent layers of novel ultra-high-efficiency solar cells at the micron and sub-micron levels.

## 1. Introduction

To effectively control the spatial thermal distribution of ever-increasing power densities in novel micro- and nano-electromechanical systems (MEMS/NEMS) and devices, more accurate temperature monitoring is needed [1,2,3]. For example, elevated channel temperatures present in novel high-electron-mobility transistors (HEMTs) can potentially cause malfunctions of the vital internal components [4,5,6,7]. Therefore, it is important to identify and analyze the local temperature variations and dynamics, not only on the device’s surface but also internally where the thermal irregularities are usually generated [8]. Similarly, in photovoltaic engineering, the proper thermal management of novel, high-efficiency, multiple-junction cell structures is crucial to reducing thermal degradation and to maintaining high and consistent energy-efficiency ratings. Thus, it would be beneficial to be able to monitor the temperature variations between individual high-energy sub-cells [9]. The corresponding challenges, however, go far beyond the established optical techniques of surface-temperature measurements, to eventually reveal three-dimensional (3D) high-resolution information about the temperature distribution in relevant materials.

Over the past decade, numerous studies have been conducted using non-invasive 2D imaging of the temperature distribution on the micron and sub-micron levels [10,11,12,13]. The most promising non-invasive measurement techniques derive from the field of luminescence thermometry, which exploits temperature-dependent spectral changes of luminescent phosphors [14,15]. In the context of this, Brites et al. [16] give a comprehensive review of the developments in luminescence thermometry, with a special emphasis on lanthanide-based thermometers and their implementation. Although reports on 3D temperature imaging also exist, their potential has yet to be fully realized. Yang et al. [17] proposed a 3D temperature-imaging system based on the localization of embedded, thermally sensitive quantum dots (QDs) for determining the temperature changes during the thermal treatment of liver-cancer cells. There are also some other techniques capable of 3D temperature imaging based on Raman thermography [18]. Micro-Raman spectroscopy, in particular, is one of the promising techniques that can provide accurate measurements of the local 3D temperature distribution in multilayer heterogeneous structures such as semiconductor devices [19,20]. Under optimal circumstances, Raman thermometry allows both high-spatial resolution and high-sensitivity temperature measurements [21,22]. Bagnall et al. [23] demonstrated a unique Raman-based technique for simultaneously probing the thermal, mechanical and electrical properties of gallium nitride HEMTs. However, due to the inherently very weak Raman signal, the corresponding thermometry can lead to inaccurate temperature measurements and misleading interpretations about heat conduction, particularly when the sample does not exhibit a suitable Raman response. Another drawback of Raman thermometry is the significant measurement error possibly arising from the short penetration depth of the laser light [24].

Recent developments in 3D temperature imaging using nano-sized fluorescence particles have found applications, mainly in life-science research [25]. However, the lack of an appropriate three-dimensional localization of nanoparticles within the specimen remains a major problem. Sokolov et al. [26] reported a novel, ultra-bright, fluorescent, ratiometric nano-thermometer that surpasses the weak brightness of existing QDs and can make 3D temperature measurements down to the nanoscale. The capabilities of the system were demonstrated using a heated wire to measure the local temperature distribution within a hydrogel embedded with nano-thermometers. Hydrogels are among the most promising candidates for thermosensitive devices for life-science applications [27,28]. Despite the encouraging results, however, efforts are still needed to increase the applicability of micro- and nano-thermometers, broadening their application into areas of microelectronics technology based on semiconductor devices.

Here, we describe the development of a fluorescence-based technique that permits non-invasive measurements of the local temperature distribution within a transparent, temperature-sensitive Er:GPF1Yb0.5Er glass-ceramic. This technique could be suited to the characterization of functionalized transparent materials that can tolerate dopant-induced changes, especially in advanced semiconductor devices. Using our previously developed experimental setup and the confocal fluorescence microscopy system [29], we performed temperature measurements based on the integrated-intensity method. By applying the electrically heated tip on top of the fluorescent sample, we were able to generate a sharp temperature gradient to make additional temperature imaging on the microscale. Moreover, by measuring the variations in fluorescence intensity along the vertical scanning path of bulk material, several thermal images were extracted and used for the reconstruction of the axial temperature distribution with a spatial resolution of a few micrometers. To verify the temperature distribution within the sample, a comparison between the experimental results and the simulation results of COMSOL Multiphysics software was performed.

## 2. Experimental Section

### 2.1. Experimental Setup

**Figure 1:**
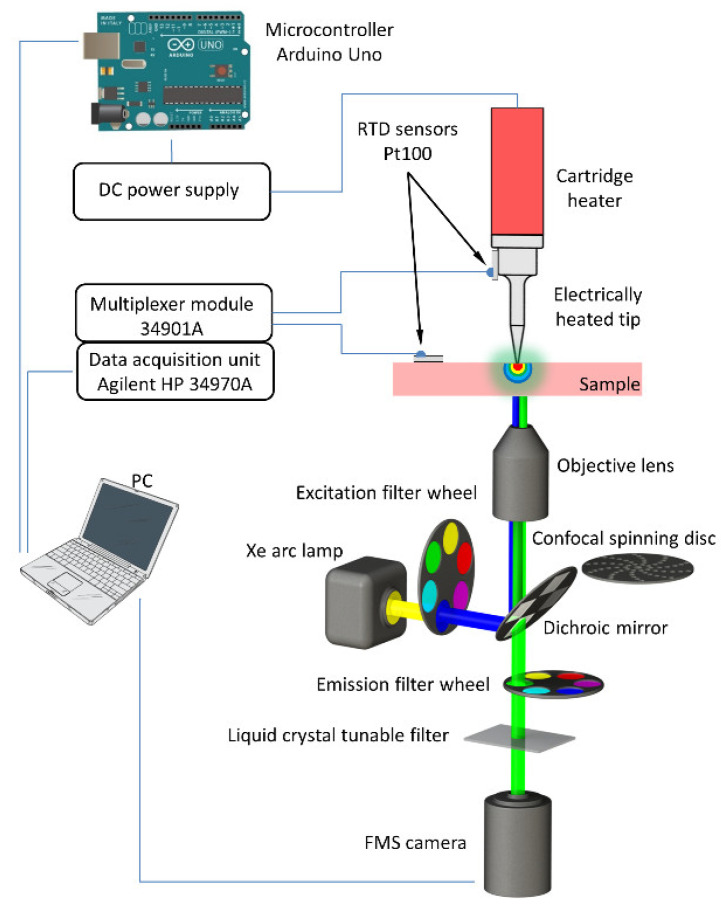
Scheme of the experimental setup. A confocal fluorescence microscopy system was used to evaluate the thermal properties of the temperature-sensitive glass-ceramic.

The experimental setup for thermal imaging of the local temperature distribution within a transparent temperature-sensitive, glass-ceramic sample is depicted in Figure 1. A sharp, electrically heated tip was mounted on top of the sample to induce highly localized heat generation at the contact surface. A computer-controlled acquisition system allowed the simultaneous triggering of the electrically heated tip, the highly sensitive (electron multiplying charge-coupled device) EMCCD camera and the temperature data-acquisition unit. The diameter of the circular contact area of the heated tip was determined with direct experimental measurements using the microscope and was found to be ~230 µm. Thermal images were acquired from different axial planes throughout the sample with the confocal fluorescence microscopy system, described in detail in our previous work [29]. The time evolution of the emitted fluorescence intensity was collected during the temperature rise until the corresponding temperature reached a quasi-steady state. Afterwards, the sample was allowed to cool down to the initial conditions at ambient temperature before proceeding to a new measurement. To ensure consistent, repeatable results during the heating cycles, the electrically heated tip was regulated with an Arduino Uno microcontroller, which enabled fast, simultaneous, and highly repeatable triggering of the EMCCD camera, the Agilent 34970A (Loveland, CO, USA) data-acquisition unit and the heated tip. In particular, the TLL signal rise time of the Arduino microcontroller was validated with a LeCroy WaveRunner 104 MXi oscilloscope and was found to be around 5 ns. Finally, two DM-314 (Labfacility, Bognor Regis, UK) miniature RTD temperature sensors were attached to the heating tip and the sample for the temperature feedback and evaluation of the fluorescent signal.

### 2.2. Fluorescence Microscopy System

As shown in Figure S1, an inverted Nikon Eclipse TE 2000-E microscope system and a CARV II (BD Biosciences, USA) fluorescence unit with a spinning disc confocal module (BD Biosciences, Franklin Lakes, NJ, USA) were used as the platform for acquiring high-resolution thermal images. As already described in Sedmak et al. [29], the glass-ceramic sample was illuminated with a 300-W Lambda LS Xe-Hg lamp (Sutter, Novato, CA, USA) in a spectral window between 430 nm and 490 nm. The temperature-dependent green emitted fluorescence light was collected with an objective lens of 40× magnification and afterwards passed through a highly sensitive Andor iXon3 897 EMCCD camera (Andor, Belfast, UK). A Varispec VIS-10-20 (CRi, Woburn, MA, USA) liquid-crystal, tunable filter (LCTF) was used for the spectral analysis and was removed during the vertical scanning of the sample. Furthermore, a Prior ProScan II (Cambridge, UK) motorized and computer-controlled XYZ-stage system was applied to ensure accurate and repeatable vertical micro-positioning of the sample.

### 2.3. Temperature-Sensing Material and Temperature Mapping

We took advantage of the thermal properties of the transparent germanate glass-ceramic co-doped with two rare-earth fluorides. This type of glass-ceramic can potentially be used in the field of non-invasive thermometry because of its excellent combination of properties, such as mechanical robustness and photo-thermal stability. The fluorescence sample was synthesized principally from a GeO_2_-PbO glassy matrix, wherein erbium fluoride (ErF_3_) and ytterbium fluoride (YbF_3_) powders were added in doping concentrations of 0.5 mol% and 1 mol%, respectively [30]. The germanium dioxide glassy matrix had the following chemical composition in mol%: 50%GeO_2_–40%PbO–10%PbF_2_–0.5%ErF_3_–1%YbF_3_.

Temperature mapping was realized through the evaluation of the relative changes in the integrated fluorescence intensity. Prior to that, axial measurements of the fluorescence intensity at a stable room temperature and fluorescence peak-intensity-ratio measurements at a single plane to determine the temperature sensitivity were performed.

Accordingly, the calibration curve was obtained by averaging the integrated emission intensity value of the 3 × 3 central pixel area of the thermal image at corresponding temperatures. Due to the weak fluorescence emission at elevated temperatures, averaging was selected to reduce the fluctuations in the temperature signal. Eventually, the temperature sensitivity was determined from the calibration curve at a broader working-temperature range from 60 °C to 150 °C and was found to be −0.6%/K at 60 °C (Figure S2) [29].

### 2.4. Numerical Simulation of the Temperature Distribution

**Figure 2:**
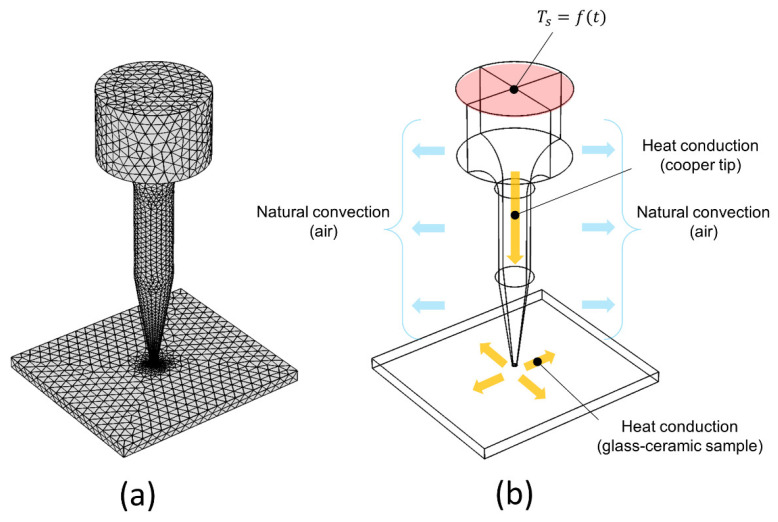
(**a**) 3D heated tip and sample model discretized by finite elements. (**b**) Schematic of the heat-transfer mechanisms.

To support our experimental measurements and for a better understanding of the transient temperature distribution at the microscale, we implemented a numerical simulation using the 3D finite-element (FE) software package COMSOL Multiphysics. In this simulation, we considered two computational domains, the electrically heated tip, and the glass-ceramic sample plate. The 3D geometry corresponding to the real heated tip and the general assembly were generated, as shown in Figure 2a. Due to the relatively simple geometry and the desired greater accuracy of the simulation, the contact layer between the heated tip and the sample was deliberately discretized into smaller elements. The contact area between the two conforming surfaces was approximately 230 µm in diameter. As represented in Figure 2b, the effect of the transient heat transfer was evaluated for the following cases: (i) heat conduction through the quickly heated copper tip, (ii) natural convection, and (iii) heat conduction through the glass-ceramic sample. Specifically, the heat was conducted through the gap between the conforming contact surfaces. The circular region indicated by the red color represents the experimentally obtained, time-dependent temperature function used as an initial temperature profile for a transient heat simulation.

The equation governing the 3D transient temperature field in a glass-ceramic sample is as follows:

(1) $$\frac{\partial }{\partial x}\left(k\frac{\partial T}{\partial x}\right)+\frac{\partial }{\partial y}\left(k\frac{\partial T}{\partial y}\right)+\frac{\partial }{\partial z}\left(k\frac{\partial T}{\partial z}\right)+\dot{q}=\rho c_{p}\frac{\partial T}{\partial t}$$

where *T* is the temperature, *ρ* is the mass density, *c_p_* is the specific heat capacity, *k* is the thermal conductivity and *t* is the time variable. We have deliberately neglected the term $\dot{q}$, which represents the internal heat generation.

The temperature on the top surface of our heated tip was set according to a time-dependent temperature-rise function determined from the experimental measurements. On the sides of the modeled heated tip and the glass-ceramic sample, we assumed heat loss due to the natural, convection-based, air-cooling mode. We predicted an average convective heat-transfer coefficient of 5 W/m^2^K for air cooling. The bulk thermal conductivity values *k* of the heated copper tip and the glass-ceramic were taken from the literature and were assigned approximate values of 401 W/mK and 0.52 W/mK, respectively [31,32]. Table 1 shows the thermophysical properties of copper and the material used in this simulation. It should be noted that due to the unknown thermal properties of the targeted bulk sample, the reference values were adopted from a comparable material. Additionally, at this stage of the study, the temperature dependence of the material properties was not considered in the simulation.

### 2.5. Image Processing

**Figure 3:**
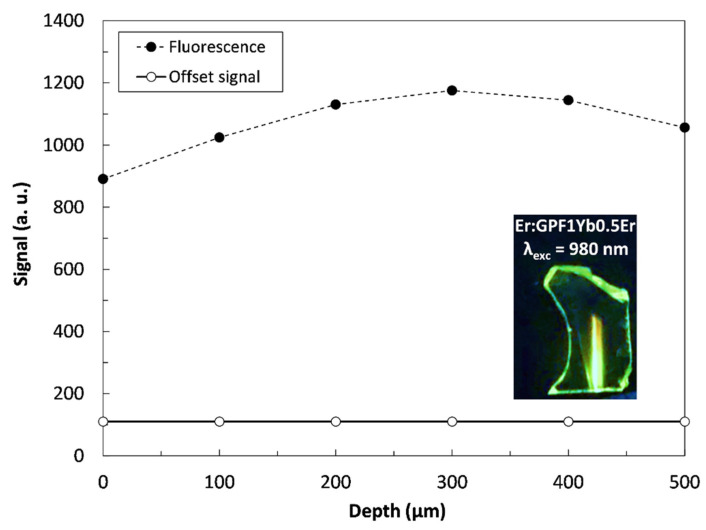
Plot of the fluorescence-intensity profile at room temperature along the *z*-axis and the offset signal from the EMCCD camera. Inset shows the green emission of a bulk sample.

The original raw images were acquired using a 16-bit grayscale format with Andor Solis acquisition software. A 40 × 0.65NA microscope configuration enabled an analyzed field of view of 190 μm × 190 μm. Individual fluorescence images with a pixel size of 512 × 512 were saved in TIF format and processed using a custom-developed MATLAB script. Afterwards, the mean gray value (MGV) was derived from the fluorescence image and correlated with the temperature-calibration curve. In the image analysis, the offset originating in the dark current and the thermal noise were first subtracted from the raw EMCCD data, after which the data was normalized with the fluorescence-intensity profile at the initial room temperature measured at different focal planes along the *z*-axis of the sample. An overall depth of 500 µm was scanned at a step size of 100 µm. As shown in Figure 3, the fluorescence intensity was most pronounced in the middle of the transparent sample.

## 3. Results and Discussion

To demonstrate the ability to image the local temperature distribution inside the transparent fluorescent material, a sharp electrically heated tip was applied to generate a micro-sized transient temperature field. Thus, increasing the temperature of the heated tip promoted the thermal dissipation into the fluorescent sample and consequently decreased the fluorescence emission’s intensity. Using the integrated-intensity calibration curve, these relative changes in the fluorescence intensity were converted to temperature values.

**Figure 4:**
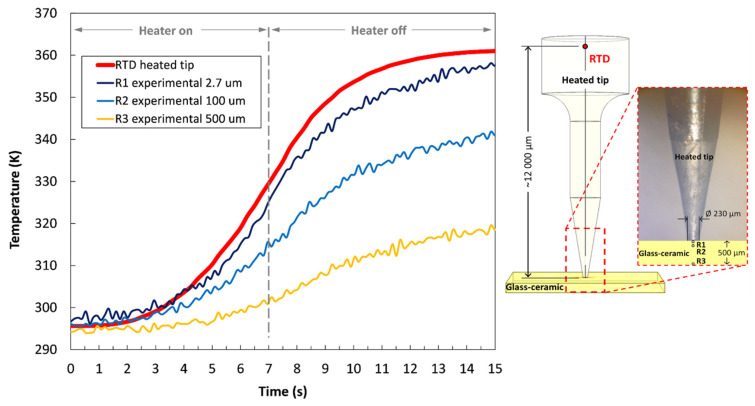
Temperature profiles measured at different axial planes within the transparent fluorescent sample during one heating cycle (**left**) using a heating tip (**right**).

During the temperature measurements, the electrically heated tip was repeatedly turned on and off with the Arduino microcontroller. It was programmed to induce heat for a duration of 7 s, with the overall acquisition time set to 15 s. An imaging frame rate of 7.5 Hz was used to effectively characterize the transient thermal behavior of the fluorescent sensing material. As presented in Figure 4, the fluorescence signal was obtained from different axial planes within the transparent sample, starting at a distance of 2.7 µm from the surface (dark blue curve) to a distance of 500 µm from the upper tip contact to the bulk (yellow curve). It is worth mentioning that the starting distance of 2.7 µm measures the temperature profile close to the surface due to the similar, micron-sized depth of field of the 40× magnification setup with NA = 0.65. The gradual thermal dissipation into the sensing material resulted in a non-uniform temperature distribution along the analyzed depth positions on the measuring optical axis. The jagged-line curves shown in Figure 4 represent the raw experimental data of the temperature-dependent fluorescence distribution within the sample at the corresponding axial planes from R1 to R3. The pronounced red curve represents the temperature response of the reference platinum RTD sensor mounted on the sidewalls of the heated tip near the cartridge heater (Figure 4, right image). As is evident from the curves, the thermal response of the RTD sensor was significant in the initial period of the heating cycle, reaching a maximum temperature difference of about 65 K. Similarly, the temperature-dependent fluorescence signal obtained from the shallower region of the fluorescent sample R1 corresponded well with the RTD response curve. This is predominantly due to the close spatial proximity between adjacent domains and due to relatively high thermal conductivity of the heated tip. When moving deeper into the sample, the transient temperature response is less pronounced, thus reducing the temperature gradient down to ~20 K in the region R3 (yellow curve). The temperature-response curves begin to flatten out close to the end of the 15 s measuring cycle, which is around (7 +/−1) s after the Arduino microcontroller automatically turns off the electric heater. Even though the electrical heater was turned off after 7 s, the temperature of the tip was still increasing because of the ongoing conductive heat transfer along the domain. This temperature lag is strongly influenced by the geometry and the material properties of the tip. Additionally, the measured temperature gradients decreased significantly when moving deeper into the sample, owing to the relatively low conductivity of the glass-ceramic itself. The accuracy of the temperature measurements is approximately ± 1.2 K, where the small random fluctuations arise from low signal-to-noise ratios, with the most noise coming from the EMCCD camera readout noise.

To better understand the local transient temperature distribution within the glass-ceramic sample, the FE model was implemented in COMSOL Multiphysics. The axial symmetry of the computed model allowed a numerical reconstruction of the 2D temperature distribution as well as plotting temperature-distribution profiles from the particular regions of interest. This would allow the current work with promising results to be expanded and, potentially, used in the field of novel transparent or semi-transparent photoelectric perovskite-like materials: for example, in analyzing degradation mechanisms or thermal anomalies that occur in multilayer devices of this kind.

**Figure 5:**
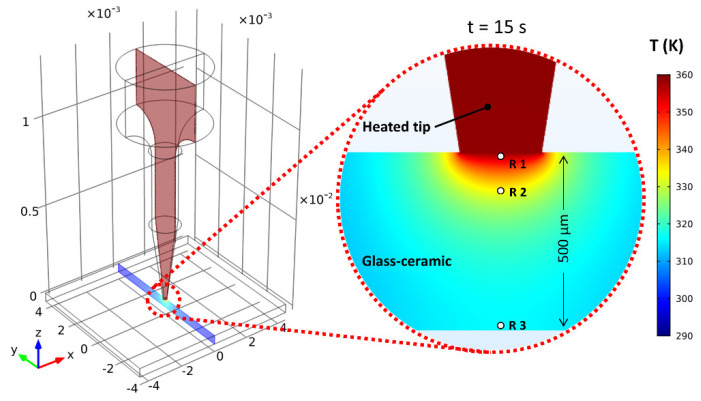
Simulated two-dimensional temperature distribution in the transparent glass-ceramic sample after the electrical resistive heating for 15 s with an electrical heater turned on for 7 s.

In our simulation, the boundary conditions considered the experimentally obtained time-dependent temperature rise function of the RTD sensor measured on the top surface of the heated tip, as previously described above. To observe the temporal evolution of the axial temperature distribution into the sample, time delays between the simulated thermal images were adjusted to match the experimental imaging conditions using a frame rate of 7.5 Hz. Figure 5 represents the simulated spatial temperature distribution in both the heated tip and the glass-ceramic sample during the electrical resistive heating. The investigation of the cross-sectional temperature distribution along the vertical axis shows a significant difference between the two domains. In particular, the temperature distribution within the heated tip was evidently more homogenous compared to the glass-ceramic sample, due to high thermal conductivity of copper. In contrast, a large temperature gradient was generated around the axial plane in the glass sample, which proved to be beneficial for thermal imaging of the transient temperature changes at relatively low frame rates.

**Figure 6:**
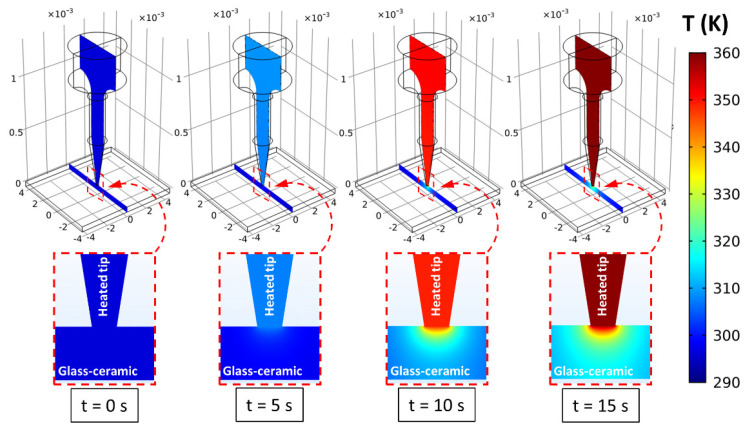
Simulated two-dimensional temperature distributions of the measuring system at different time points.

Figure 6 shows the simulated temperature distribution images in the region of interest with respect to different time points. During the conductive heat transfer along the domains, a large temperature increase is clearly seen in the magnified inset of the figure. The results indicate that the corresponding optical technique can potentially be adopted for precise spatial scanning of the transient temperature distribution within transparent solid bulk materials with low thermal conductivity.

**Figure 7:**
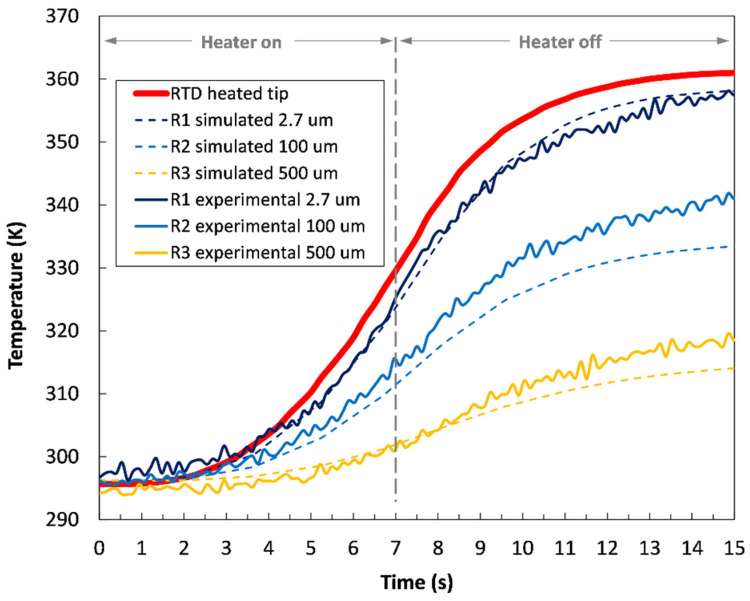
Experimental and corresponding simulated vertical temperature profiles measured at different axial planes within the transparent fluorescent sample.

Simulated axial temperature profiles at the corresponding measured depths are presented in Figure 7 with the dashed curves. The curves bear a strong resemblance to the experimental results, even at the farthest simulated location R3 at −500 µm. A good agreement of the simulated profiles, as well as their observed trends, with the experimental data is shown by only changing the depth parameter, which means that the process is repeatable and thus the model is well justified. The simulated temperature distribution tends to flatten after having passed the inflection point at 7 s, which is in accordance with the experimental results. As stated previously, the simulated temperature profiles tend to diverge slightly compared to the experimentally obtained profiles. The reason for this could be related to the adopted bulk thermal conductivity of the fluorescent material described in Section 2.4 and to the potential thermal expansion of the experimental test section assembly. These conditions can result in uneven specimen heating and associated focal drifting. Nevertheless, the research shows that under controlled conditions this technique successfully enables robust measurements of the axial temperature distribution across the sample with high spatial resolution.

In our case, a 3D reconstruction of the transient temperature distribution could also be possible, but due to high-magnification imaging of the field of view having a similar size to the contact area between the heating tip and the glass sample, no particular lateral information about the temperature distribution could be gained. For a high-resolution 3D reconstruction, new micron-sized heating systems are required, and are under development.

## 4. Conclusions

Due to ever-increasing demands to characterize the temperature distributions within semiconductor devices, advanced measurement techniques such as fluorescence microscopy offer an important engineering tool for their testing, which is essential for designs that improve the reliability. In this article, we adopted the fluorescence-based technique for non-invasively measuring the local temperature distribution inside the transparent glass-ceramic as a model for measurement and control in advanced manufacturing. Our experimental technique relied on a stable, temperature-dependent fluorescence of the germanate glass-ceramic co-doped with rare-earth fluorides. Using a sharp, electrically heated tip mounted on top of the glass-ceramic material for the local heat generation of micron-sized transient temperature fields and a confocal fluorescence microscopy system, we observed the temperature distribution at different focal planes across the 500-µm-thick temperature-sensitive sample. Thermal images were obtained directly below the contact surface of the corresponding domains, at distances of 2.7 µm, 100 µm and 500 µm. This was achieved by measuring the temperature dependency of the fluorescence intensity over the emission spectra typical for the used glass-ceramic. Furthermore, the application of non-invasive temperature measurements allowed us to reconstruct the axial temperature distribution within the sample during a heating cycle. The results showed good agreement between the experimental results and simulated thermal images based on finite-element analyses.

To further improve the spatial resolution of the temperature measurements, a more rigid positioning system for the heated tip would be required, along with a high-numerical-aperture immersion objective lens with a greater magnification than 40×. Moreover, a custom-made temperature-controlled microscope probe chamber, integrated within the existing computer-controlled XYZ-stage system could be beneficial, as it would substantially shorten the cooling cycle between consecutive measurements, as well as improving the signal-to-noise ratio of the fluorescence. A new, micron-sized heating source system is also under development for high-resolution 3D reconstruction. We also anticipate that the corresponding fluorescence technique with further technological improvements will find uses in many areas of science, especially in the non-invasive submicron thermal characterization of future solid-solid or even solid-liquid interfaces and the corresponding devices.

## Figures and Tables

**Table 1:** Material properties used in COMSOL Multiphysics.

Material	Density, kg/m^3^	Thermal Conductivity, W/(m∙K)	Specific Heat, J/(kg∙K)	Refs.
Copper	8920	401	380	[31]
Reference material	5800	0.52	320	[32,33,34]

## Data Availability

Not applicable.

## Supplementary Materials

The following are available online at: https://www.mdpi.com/article/10.3390/s22051970/s1. Figure S1: Photographs of the fluorescence microscopy system; Figure S2: (a) Fluorescence emission spectra versus temperature of the Er:GPF1Yb0.5Er glass-ceramic. The inset figure shows the temperature dependence of the fluorescence peak-intensity ratio I_525_/I_547_ during the heating and cooling cycle. (b) Calibration curve obtained by the integrated-intensity method.
