# Supplementary figures and images for: Spatially Resolved Temperature Distribution in a Rare-Earth-Doped Transparent Glass-Ceramic
Source: Sensors (Basel). 2022 Mar 2;22(5):1970. doi: 10.3390/s22051970 (PMC8914839; doi:10.3390/s22051970)

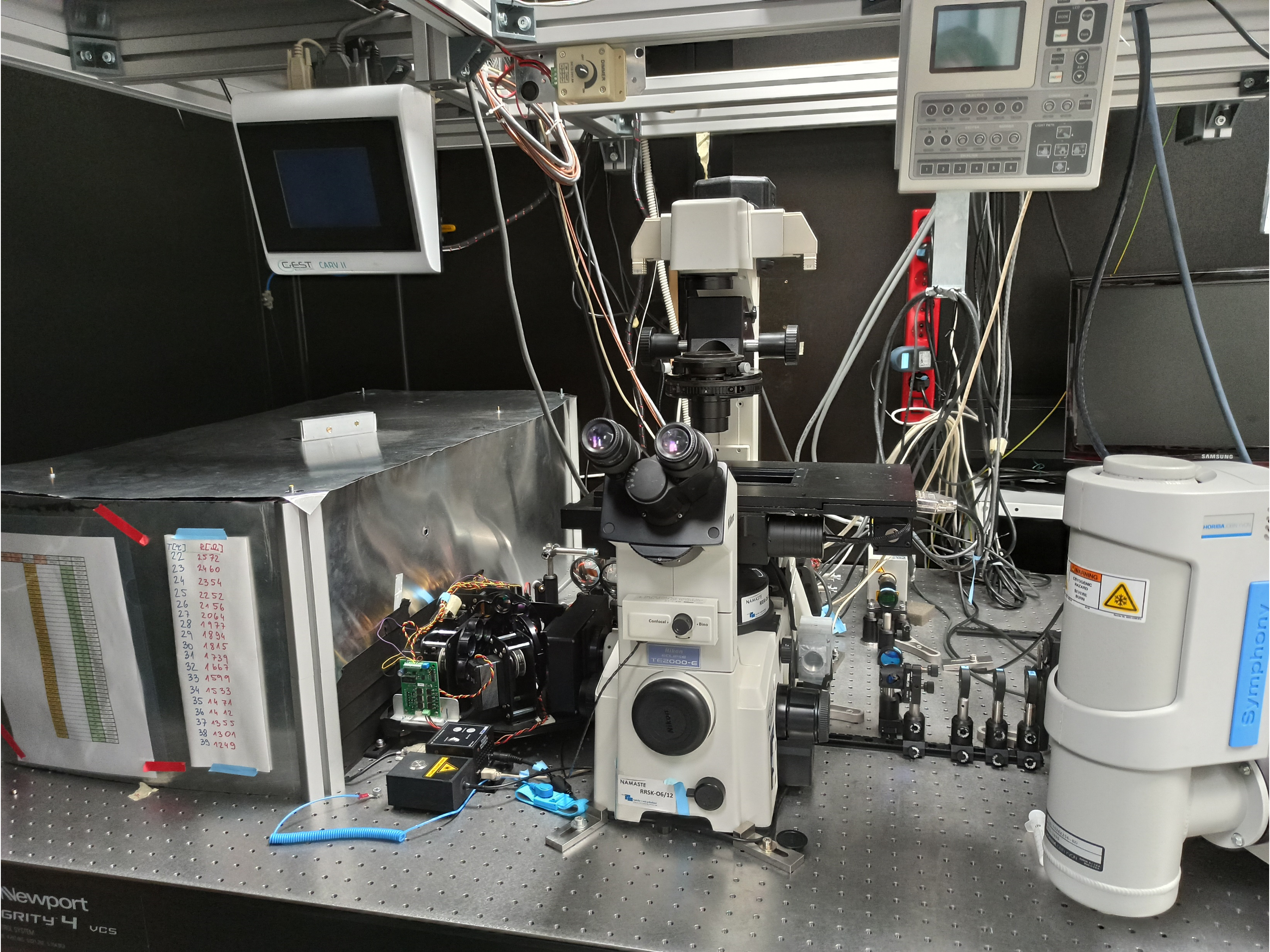

Supplement: Supplementary file 1 [file sensors-22-01970-s001.zip › Figure S1.tif]

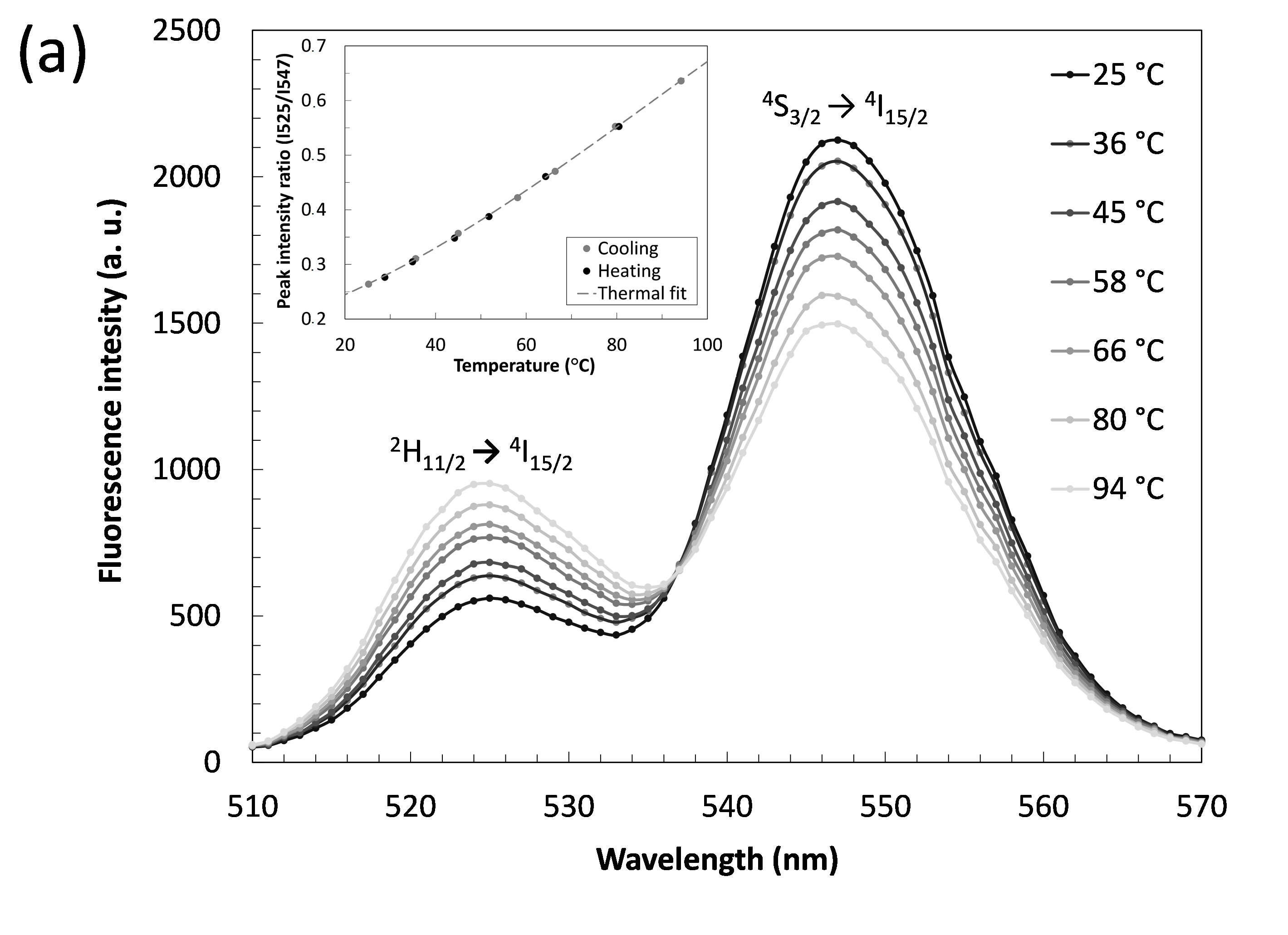

Supplement: Supplementary file 1 [file sensors-22-01970-s001.zip › Figure S2a.tif]

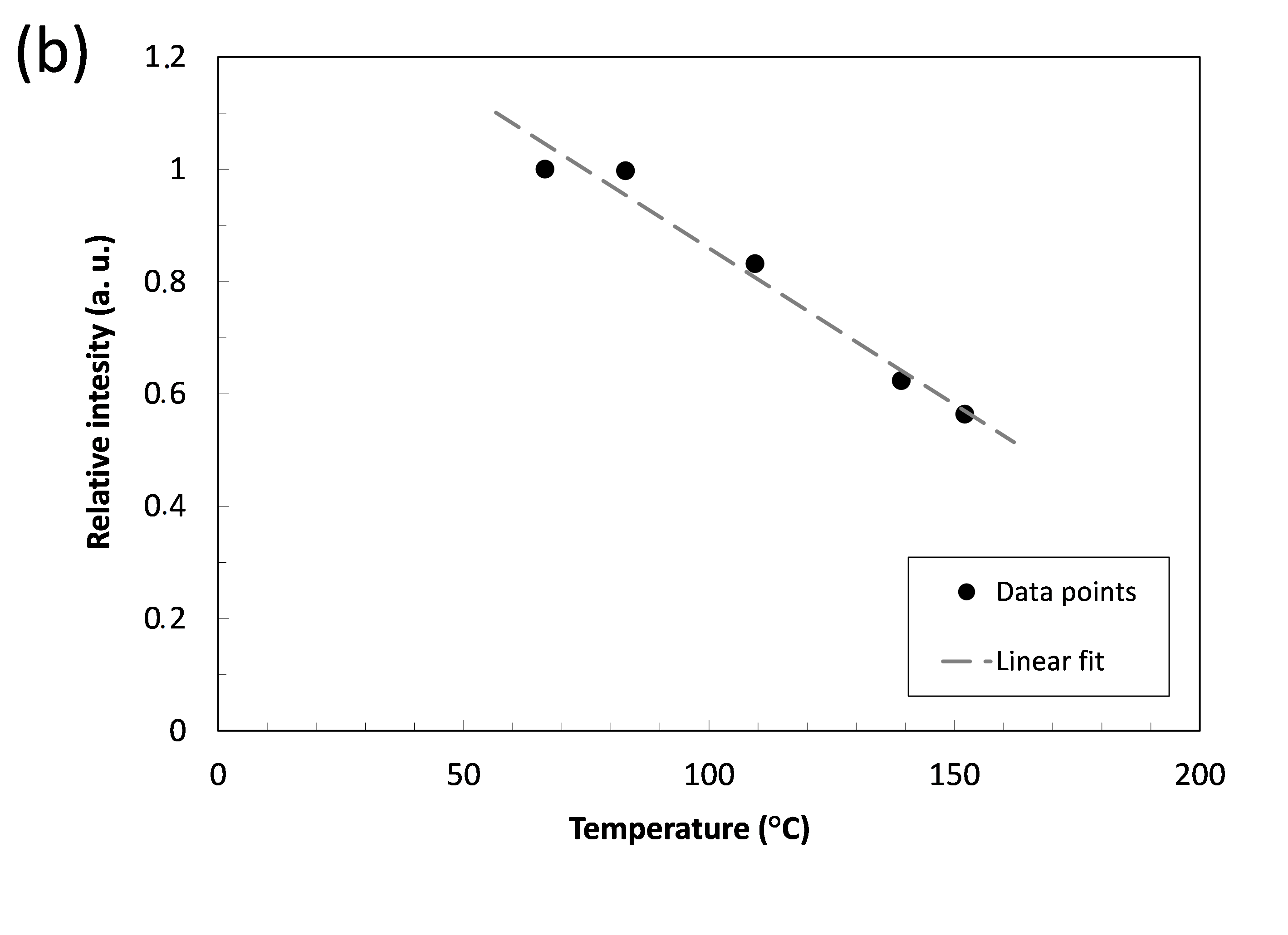

Supplement: Supplementary file 1 [file sensors-22-01970-s001.zip › Figure S2b.tif]
